# Gestational age, birth weight, and infant 1-year mortality risk: A large-scale analysis of 6.9 million births from the Japanese National registry

**DOI:** 10.1371/journal.pone.0335191

**Published:** 2025-10-30

**Authors:** Miho Sassa, Ayaka Monoi, Yayoi Murano, Utako Kondo, Hiromichi Shoji, Daisuke Yoneoka

**Affiliations:** 1 Department of Global Health Policy, Graduate School of Medicine, The University of Tokyo, Tokyo, Japan; 2 National Institute of Infectious Diseases, Japan Institute for Health Security, Tokyo, Japan; 3 Department of Pediatrics, Juntendo University, Faculty of Medicine, Tokyo, Japan; 4 Department of Neonatology, Tokyo Metropolitan Bokutoh Hospital, Tokyo, Japan; Kobe University Graduate School of Medicine School of Medicine, JAPAN

## Abstract

**Background:**

Japan’s low infant mortality rates (IMR) reflect advancements in neonatal care. However, a comprehensive understanding of factors influencing long-term infant mortality nationwide is lacking. We examined the 1-year relationships between gestational age (GA), birth weight, small-for-gestational-age (SGA) status, and infant mortality risk.

**Objective:**

This study aimed to estimate the 1-year survival probability of infants stratified by GA, birth weight and SGA status.

**Methods:**

This study is an observational study using Japan’s vital registration data between 2012 and 2018. Whole national birth and death registration data was analyzed. 6,918,305 births and 12,440 deaths within the first year of age were included. The main outcome is 1-year infant mortality and survival curve.

**Results:**

Infants born preterm, and SGA exhibited elevated mortality rates (15 and 8.5 per 1,000, respectively). Survival probability varied significantly based on GA, birth weight, and SGA status. Overall 1-year mortality risk was 1.8 per 1,000 live births with survival probabilities notably increasing with GA. Late preterm and early term births also exhibited increased mortality risks compared to full term infants (e.g., 2.2 per 1,000 live births if born at 37 weeks vs 0.76 at 39 weeks). Notably, late preterm births constituted the majority of preterm births and were associated with major mortality risks.

**Conclusions:**

This study, extending beyond previous short-term research, provides insights into long-term infant mortality risks in Japan, underscoring the importance of gestational age, birth weight, and SGA status in infant mortality risk among live births. Further study to elucidate the reasons behind pregnancy termination decisions and the specifics of antenatal and neonatal care is needed to better understand the factors influencing infant mortality.

## Introduction

Neonatal mortality rate (NMR) and infant mortality rates (IMR) serve as traditional indicators of the quality of healthcare and social conditions across countries [1,2]. In 2021, the most recent year with available data for all OECD member nations, Japan reported an IMR of 1.7 per 1,000 live births, which is notably lower than the OECD member nations’ average of 4.0 per 1,000 live births. Japan secured the 2nd position among the 34 OECD member countries in terms of IMR [3].

The survival rate of infants including those born preterm has been remarkably improved in recent decades, largely due to advancements in perinatal care and obstetrics management for high-risk mothers and treatment interventions for preterm infants [4]. The most significant breakthrough occurred in the 1980s with the introduction of pulmonary surfactant, which notably increased survival rates among preterm infants with respiratory distress [5]. The clinical use of surfactant was associated with a marked change in their clinical trajectory [6]. Other therapeutic interventions such as ventilation techniques [7], antenatal glucocorticoid therapy, aggressive nutrition policies, and appropriate oxygen supplementation continue to play vital roles in improving outcomes for preterm infants [8] Due to these achievements, in the United States, from 1980 to 1993, the survival rate of infants born weighing between 500 g and 550 g improved significantly from 35% to 70% [9]. In Germany, by 2010, 90% of infants born with very low birth weight (i.e., birth weight <1500 g) survived [10]. Japan, renowned for its excellence in neonatology, has also witnessed improvements in mortality rates among infants with very low birth weights from 2003 to 2008. During this period, mortality decreased from 10.8% to 8.7% among infants born with weights ranging from 501 g to 750 g [11]. Remarkably, decreases in mortality were observed even among infants weighing less than 400 g [12], underscoring significant advancements in neonatal care and interventions aimed at improving outcomes for the most vulnerable newborns.

In addition, the incidences of preterm birth and small-for-gestational-age (SGA) in infants are still problematic. We previously reported that gestational age and SGA are independent risk factors associated with body mass index [13]. The SGA rate varies considerably depending on regions, with higher rates observed in developing countries [14,15]. While the global preterm birth rate is approximately 10% [15], Japan reports a lower rate [16,17]. However, preterm birth remains a risk factor for perinatal and infant mortality, as well as adversely affecting neurodevelopment and increasing the incidence of cardiovascular diseases [18]. In recent years, 37–38 week term births, categorized as ‘early term’ by the American College of Obstetricians and Gynecologists (ACOG) in 2013 [19], have garnered increasing attention. These births exhibit a higher rate of neonatal complications and mortality compared to the 39–41 week term births [19,20]. While there have been sporadic reports of elevated risks of neonatal complications and mortality in early term newborns (37–38 weeks) compared to full term babies (39–41 weeks), there is a scarcity of studies reporting on the prognosis of early term babies in Japan.

Infants born with SGA, another adverse birth outcome that is the focus of epidemiological studies, face an elevated risk of delayed development or childhood mortality [21]. Previous studies have demonstrated significant improvements in mortality and morbidity among extremely low birth weight and very preterm infants in Japan [22]. These studies primarily focused on newborns admitted to neonatal intensive care units (NICUs), with outcomes assessed in terms of early neonatal mortality rate (<7 days of age), neonatal mortality rate (<28 days of age), and mortality rate during NICU stay. However, there is limited knowledge regarding long-term outcomes such as infant mortality among those discharged from NICUs and newborns who were not admitted to NICUs. We conducted a population-based study of infant survival rates to assess factors associated with long-term infant mortality.

## Materials and methods

Birth and death certificate data were collected from the Ministry of Health, Labor, and Welfare in Japan’s vital registration statistics from January 1st, 2012 to December 31st, 2018, for analysis. In addition to the individual’s birthday, birthweight and gestational age (GA) were also extracted. Infants were categorized by birth weight: those born weighing < 1,000g, 1,000–1,499g, 1,500–2,499g, 2,500–3,999g, and ≥4,000g. In addition, every infant was also categorized by GA: extremely preterm (22–27 weeks), very preterm (28–31 weeks), moderate to late preterm (32–36 weeks), term (37–41 weeks), and post term births (42–44 weeks). Then, we defined SGA as infants born under 10 percentile birth weight at each GA value [23]. The analysis included live births born at ≥ 22 and <45 GA weeks. Infant mortality within the first year of life was analyzed with respect to GA, birth weight, and the presence of SGA. Kaplan-Meier survival curves were constructed to estimate the survival probability based on GA, birth weight, and SGA status and compared using log-rank tests. We aggregated the live birth data and the death data and used the live birth data as censoring in constructing the Kaplan-Meier survival curves. The confidence interval (CI) of IMR was constructed by binomial exact method. To minimize the influence of confounding factors, we also conducted a Cox regression analysis. In this analysis, we adjusted for the available variables including maternal age, infant sex, gestational age, birth weight, plurality, calendar month of birth date, and prefecture. Statistical analysis was performed with R version 4.3.2. In this paper, statistical significance was defined as a p-value less than 0.05.

## Results

Among 6,919,379 births from 2012 to 2018, a total of 6,918,305 births (99.98%) with GA recorded as 22–44 weeks were included in this study, while 34 births (0.00049%) recorded GA outside this range and 1,040 births (0.015%) with missing GA were excluded. Among 13,373 deaths within the first year of age, 12,440 deaths (93.0%) with GA recorded as 22–44 weeks were included in this study and 907 deaths (6.8%) were excluded due to lack of GA information. Among all the births included in this analysis, the mean GA was 38.7 (25–75% quantiles: 38–40) weeks and the mean birthweight was 3,002g (25–75% quantiles: 2,762–3,272g). Among these infants, there were 3,547,827 (51.3%) males. Among these infants, 391,646 (5.7%) infants born preterm, 656, 697 (9.5%) were low birth weight, and 486,817 (7.0%) were SGA. Among infants born during 2012–2018, the early neonatal mortality rate (<7 days of age) was 0.67 per 1,000 live births, the neonatal mortality rate (0 to 27 days of age) was 0.91 per 1,000 live births, and the infant 1-year mortality rate was 1.8 per 1,000 live births. Supplemental Table 1 summarizes the infant mortality rate by GA, birth weight, and presence of SGA.

**Fig 1:**
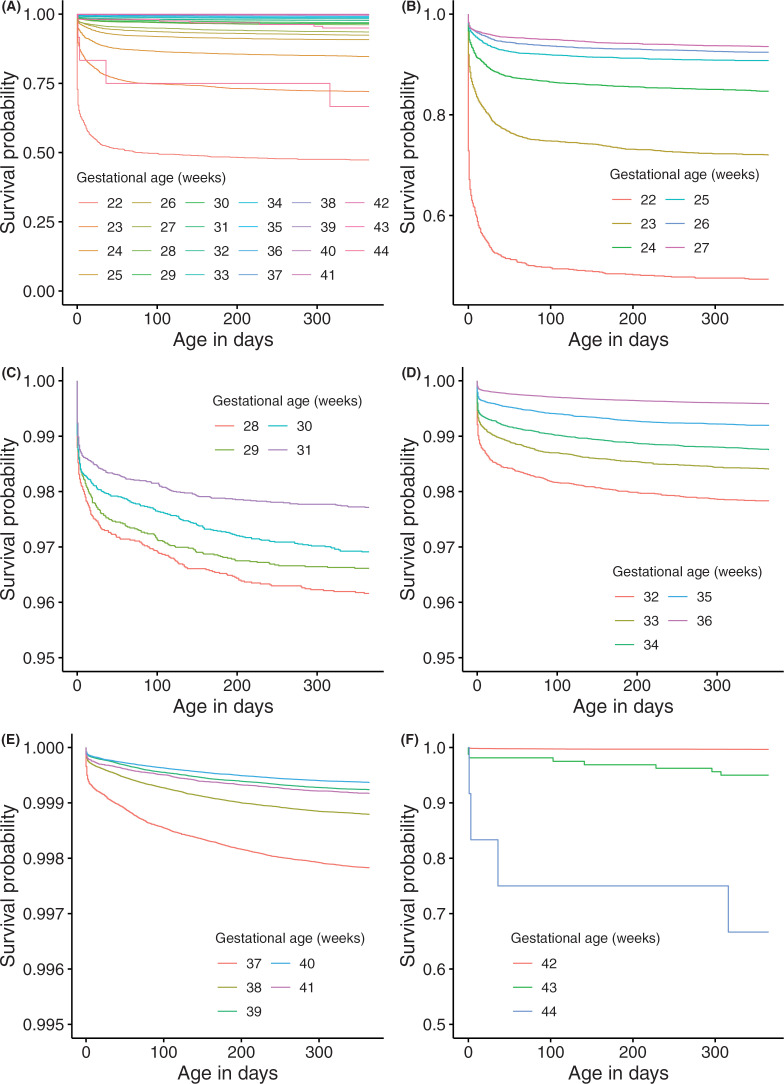
Survival curves of live births by gestational age week between 2012 and 2018. (A) 22-44 gestational age weeks, (B) Extremely preterm birth, (C) Very preterm birth, (D) Moderate to late preterm birth, (E) Term birth, (F) Post-term birth.

**Fig 2:**
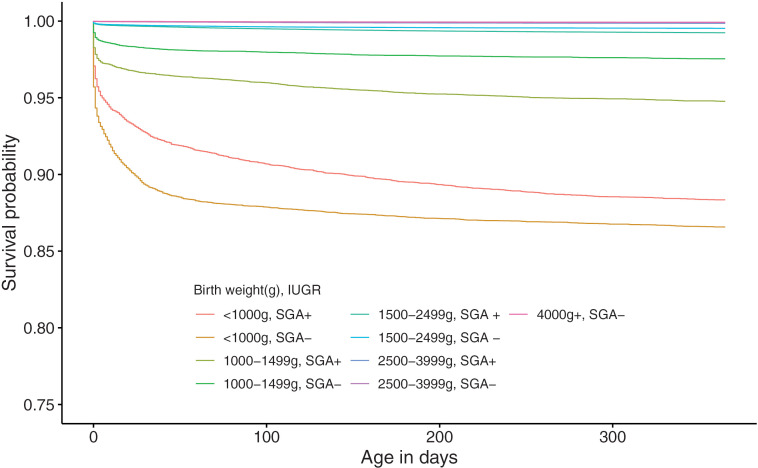
Survival curves of live births by birth weight category and status of small-for-gestational-age between 2012 and 2018.

**Fig 3:**
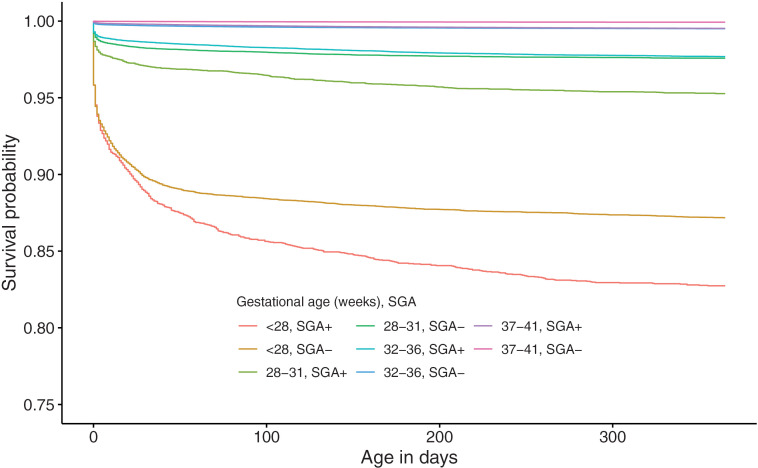
Survival curves of live births by gestational age category and status of small-for-gestational-age between 2012 and 2018.

Fig 1A shows the cumulative survival rates up to 365 days after birth of infants, stratified by GA from 22 weeks to 44 weeks. The Kaplan-Meier survival curve for extremely preterm births (GA between 22 and 27 weeks) infants exhibited a steady decline in survival probability within the first 30 days (Fig 1B). The survival rate at 365 days post-birth among extremely preterm births notably improved as GA increased. Infants born at 22 weeks of GA had a significantly low survival probability of 47.4% (95% CI: 44.1 - 50.6) at 365 days post-birth. The survival rate among very preterm births (GA between 28 and 31 weeks) was over 96% at 365 days post-birth (Fig 1C). Among moderate to late preterm births (GA between 32 and 36 weeks), it was observed that the infant survival rate clearly increases with each additional week of GA (Fig 1D). Among term births (GA between 37 and 41 weeks), the survival rate of newborns born at 37 and 38 weeks of GA was significantly lower (37 weeks vs 38 weeks, p < 0.001; 38 weeks vs 39 weeks, p < 0.001) (Fig 1E). The survival curve showed that newborns born at 44 weeks of GA had lower survival probability than those born at 42 and 43 weeks of GA (Fig 1F). The log-rank test revealed that the cumulative survival rate significantly increased as birthweight increased up to the 2,500−3,999g range (1,500–2,499g vs 2,500–3,999g, p-value <0.001), but there was no difference in the cumulative survival rate above the 4,000g range (2,500–3,999g vs ≥ 4,000g, p-value = 0.6). Similarly, the cumulative survival rate was significantly higher in the larger GA group up to 28 weeks of GA (e.g., 27 weeks vs 28 weeks, p-value < 0.001), however, there was no statistically significant difference at some GA weeks above 28 weeks of GA (e.g., 28 weeks vs 29 weeks, p-value = 0.2). Regarding the presence of SGA, the cumulative survival rate was significantly higher in non-SGA infants (e.g., in the 1,500−2,499g group, p-value < 0.001). In the < 1,000g group, the cumulative survival rate was significantly higher in SGA infants (SGA vs non-SGA, p-value < 0.001) (Fig 2). Fig 3 showed the cumulative survival rates up to 365 days after birth of infants, stratified by GA subgroup (between 22 and 27 weeks, between 28 and 31 weeks, between 32 and 36 weeks, between 37 and 41 weeks) and the presence of SGA. Note that, for infants born between 42 and 44 weeks, SGA is not defined. Among SGA infants, the cumulative survival rate was significantly lower in earlier GA subgroups. We also found that the hazard ratios for gestational age and birth weight in the Cox regression model are 0.968 (p-value < 0.001) for gestational age and 0.998 (p-value < 0.001) for birth weight.

## Discussion

Our study elucidates the infant mortality rates in Japan, utilizing a population-based registry database from 2012 to 2018. We have estimated an infants’ 1-year survival curve specific to birth weight, GA, and presence of SGA. Infants born preterm, post-term, SGA, and males exhibit a heightened mortality rate. These identified risk factors align with existing literature findings [24,25].

Late preterm birth constituted the majority of preterm births and was associated with a smaller but statistically significant risk of neonatal mortality and morbidity [26]. In our study, late preterm birth accounted for 79.5% of all preterm births, which was higher than the proportion observed in the United States (71.0%) [27]. Compared with the infant mortality rate at 39 weeks of gestation, late preterm newborns faced 16.3-, 10.6-, and 5.4-fold increased risks of death at 34 weeks, 35 weeks, and 36 weeks of gestation, respectively. Even among term neonates, the infant mortality rate was higher among those born at 37 weeks and 38 weeks of gestation (early term) compared with those born at 39 weeks of GA. The relative risk was 2 to 4 for infant mortality in our study and the reported literature [27]. Consequently, the American College of Obstetricians and Gynecologists Committee suggested that although there were specific indications for delivery prior to 39 weeks of gestation, a non-medically indicated early-term delivery was not appropriate [28].

Our study also demonstrated a very low infant mortality rate between 2012 and 2018 in Japan. Effective medical care services, including routine prenatal care and testing, interventions to mitigate the mortality and morbidity of preterm infants, and the affordable cost of intensive care, may contribute to this improvement. Over the past decades, infant mortality in Japan has exhibited a decreasing trend: from 30.7 per 1,000 live births in 1960, to 13.1 per 1,000 in 1970, to 7.5 per 1,000 in 1980, to 2.3 per 1,000 in 2010, and 1.7 per 1,000 in 2020 [3]. This trend aligns with the global pattern observed in the Global Burden of Disease 2013 Study [29]. Previous studies have demonstrated that extrauterine growth restriction is a well-known complication observed at discharge among infants born preterm, which is associated with cardiometabolic and neurodevelopmental outcomes [30]. Other studies have suggested that morbidities including gastroesophageal reflux, eating disorders, sleeping disorders, and abnormal fat distribution are also associated with growth among infants born preterm [30,31]. Therefore, further investigation with long-term follow-up is warranted from this perspective.

Neonates born with GA less than 32 weeks exhibit proportionately high mortality risks compared to term neonates in general [24,25]. To better understand determinants of survival rates in extremely preterm infants, antenatal and neonatal care practices as well as morbidity need to be elucidated at the facility level. Furthermore, there may be heterogeneity in the criteria for registering neonates born at 22 weeks of gestation as live births, which is out of the scope of our analysis. Although mortality is a critical and immediate outcome, we acknowledge that long-term functional outcomes, including neurodevelopmental and cardiometabolic sequelae, are essential to fully understand the impact of gestational age, birth weight, and the presence of SGA. However, our dataset does not include such clinical outcomes, and we were unable to assess these longer-term outcomes. Future research using clinical registries or longitudinal cohort data is required to investigate how early-life factors influence health trajectories across the life course.

This study also has several other limitations. The first limitation pertains to the large number of infants with missing data. This may result in the overestimation of the survival probability. However, the proportion of missing data in the dataset is less than 10 percent, which is not likely to change the results drastically. Moreover, we utilized national death registration data, which represents the most comprehensive database covering the entire population. The second limitation is that we did not consider the cause of death and multiple pregnancies in our analysis, thus limiting our ability to establish causality and adjust for potential confounding factors, including fatal deformities. The third limitation is that we did not consider reasons for terminating pregnancy, which are likely to determine gestational ages. While our findings are based on a comprehensive national database with high registration accuracy, important clinical variables such as maternal complications, mode of delivery, NICU admission, and treatment intensity were not available. As such, residual confounding cannot be entirely adjusted regardless of the use of the Cox regression. Future studies incorporating more detailed clinical data are required to examine further the mechanisms linking perinatal factors with neonatal outcomes. The fourth limitation is that we did not assess the severity of SGA status; instead, we only determined the presence or absence of this condition. However, in our clinical practice, the presence of SGA is used as a clinical marker rather than its severity. Therefore, understanding the long-term outcomes associated with birthweight, gestational week, and SGA may hold greater clinical relevance than assessing the associations between the severity of growth restriction and mortality. Lastly, our analysis revealed that among infants with birth weight under 1000g, those classified as SGA demonstrated a higher survival rate compared to non-SGA infants. Importantly, our dataset does not include information on clinical details, such as anomalies among live births, mode of delivery, or NICU interventions. Future studies using hospital-based or cohort-level clinical data are needed to explore these mechanisms or further confounding in greater detail. In addition, while we acknowledge that the analysis of cause-specific mortality or long-term functional outcomes would offer further clinical insight, such analyses were beyond the scope of this study due to data limitations. Specifically, although causes of death such as congenital anomalies are recorded in the mortality database, corresponding information is not available in the birth registry. As a result, we cannot identify underlying health conditions in infants who survived, making it difficult to conduct analyses stratified by comorbidities or specific causes. Future studies using datasets that integrate both perinatal clinical information and long-term follow-up are warranted to address these important questions.

## Conclusion

This study provides valuable insights into long-term infant mortality risks in Japan, highlighting the complex interplay of risk factors such as GA, birth weight, and SGA status. By examining long-term mortality outcomes for conditions such as SGA, our findings enable comparisons with other diseases, contributing to a more comprehensive understanding of the relative impact of various health conditions on infant survival. Furthermore, this study extends beyond the scope of previous short-term infant mortality research, revealing the long-term consequences of perinatal factors that may not have been apparent in earlier studies. Despite Japan’s remarkable progress, ongoing nationwide studies are needed to better understand mortality risks among diverse birth outcomes, and targeted strategies are crucial for sustaining advancements in infant health. Collaborative efforts involving healthcare professionals, policymakers, and researchers can drive interventions that consider both short-term and long-term perspectives to further reduce infant mortality rates and improve outcomes nationwide.

## Supporting information

Table S1 Infant mortality by gestational age and birth weight between 2012 and 2018. (DOCX)
