# Supplementary material for: Gestational age, birth weight, and infant 1-year mortality risk: A large-scale analysis of 6.9 million births from the Japanese National registry
Source: PLoS One. 2025 Oct 30;20(10):e0335191. doi: 10.1371/journal.pone.0335191 (PMC12574836; doi:10.1371/journal.pone.0335191)
Supplement: Table S1 — (DOCX) [file pone.0335191.s001.docx]

**Supplementally Table 1: Infant mortality by gestational age and birth weight between 2012 and 2018**

|  |  | **Number of live births** | **Number of deaths in less than 1 year infants** | **Mortality in less than 1 year per 1000 live births** | **95% Confidence Interval** |
| --- | --- | --- | --- | --- | --- |
| **Total** |  | 6918305 | 12440 | 1.80 | (1.77, 1.83) |
| **Sex** | Female | 3370478 | 5842 | 1.73 | (1.69, 1.78) |
|  | Male | 3547827 | 6598 | 1.86 | (1.82, 1.91) |
| **Gestational age (week)** | |  |  |  |  |
|  | 22 | 925 | 487 | 526.49 | (493.73, 559.08) |
|  | 23 | 2188 | 612 | 279.71 | (260.98, 299.03) |
|  | 24 | 2756 | 422 | 153.12 | (139.87, 167.11) |
|  | 25 | 3193 | 294 | 92.08 | (82.27, 102.64) |
|  | 26 | 3793 | 287 | 75.67 | (67.45, 84.55) |
|  | 27 | 4505 | 290 | 64.37 | (57.38, 71.94) |
|  | 28 | 5807 | 223 | 38.40 | (33.61, 43.67) |
|  | 29 | 6586 | 223 | 33.86 | (29.62, 38.52) |
|  | 30 | 8549 | 264 | 30.88 | (27.32, 34.77) |
|  | 31 | 10762 | 246 | 22.86 | (20.12, 25.86) |
|  | 32 | 15646 | 339 | 21.67 | (19.44, 24.07) |
|  | 33 | 22166 | 353 | 15.93 | (14.32, 17.66) |
|  | 34 | 38337 | 476 | 12.42 | (11.33, 13.58) |
|  | 35 | 69736 | 561 | 8.04 | (7.39, 8.74) |
|  | 36 | 196697 | 810 | 4.12 | (3.84, 4.41) |
|  | 37 | 748429 | 1624 | 2.17 | (2.07, 2.28) |
|  | 38 | 1505925 | 1816 | 1.21 | (1.15, 1.26) |
|  | 39 | 1940523 | 1476 | 0.76 | (0.72, 0.80) |
|  | 40 | 1744328 | 1099 | 0.63 | (0.59, 0.67) |
|  | 41 | 572247 | 473 | 0.83 | (0.75, 0.90) |
|  | 42 | 15035 | 53 | 3.53 | (2.64, 4.61) |
|  | 43 | 160 | 8 | 50.0 | (21.83, 96.14) |
|  | 44 | 12 | 4 | 333.33 | (99.25. 651.12) |
| **Birth weight (g)** | |  |  |  |  |
|  | <1000 | 20798 | 2632 | 126.55 | (122.06, 131.15) |
|  | 1000-1499 | 30905 | 1150 | 37.21 | (35.13, 39.38) |
|  | 1500-2499 | 604994 | 3670 | 6.07 | (5.87, 6.27) |
|  | 2500-3999 | 6206521 | 4931 | 0.79 | (0.77, 0.82) |
|  | 4000+ | 550887 | 47 | 0.85 | (0.63, 1.13) |
|  | NA | 0 | 10 | - |  |
| **Small-for-gestational-age** | |  |  |  |  |
|  | + | 486817 | 4147 | 8.52 | (8.26, 8.78) |
|  | - | 6412656 | 8219 | 1.28 | (1.25, 1.31 ) |
|  | NA | 18832 | 74 | - |  |
